# Designing Quantum Spin-Orbital Liquids in Artificial Mott Insulators

**DOI:** 10.1038/srep31737

**Published:** 2016-08-24

**Authors:** Xu Dou, Valeri N. Kotov, Bruno Uchoa

**Affiliations:** 1 Department of Physics and Astronomy, University of Oklahoma, Norman, OK 73069, USA; 2 Department of Physics, University of Vermont, Burlington, VT 05405, USA; 3 Department of Physics and Astronomy, University of Oklahoma, Norman, OK 73069, USA

## Abstract

Quantum spin-orbital liquids are elusive strongly correlated states of matter that emerge from quantum frustration between spin and orbital degrees of freedom. A promising route towards the observation of those states is the creation of artificial Mott insulators where antiferromagnetic correlations between spins and orbitals can be designed. We show that Coulomb impurity lattices on the surface of gapped honeycomb substrates, such as graphene on SiC, can be used to simulate SU(4) symmetric spin-orbital lattice models. We exploit the property that massive Dirac fermions form mid-gap bound states with spin and valley degeneracies in the vicinity of a Coulomb impurity. Due to electronic repulsion, the antiferromagnetic correlations of the impurity lattice are driven by a super-exchange interaction with SU(4) symmetry, which emerges from the bound states degeneracy at quarter filling. We propose that quantum spin-orbital liquids can be engineered in artificially designed solid-state systems at vastly higher temperatures than achievable in optical lattices with cold atoms. We discuss the experimental setup and possible scenarios for candidate quantum spin-liquids in Coulomb impurity lattices of various geometries.

Quantum spin liquids are highly entangled states that can emerge in antiferromagnetic lattices in the presence of spin degeneracies and frustration. Spin-orbital liquids result from systems that have not only spin degeneracies but also orbital degeneracies^1^,^2^. Those states are strongly correlated, have non-local excitations, but nevertheless do not break any symmetries. In spite of mounting theoretical effort^3^,^4^,^5^,^6^,^7^, a significant difficulty in finding viable candidates for quantum spin-orbital liquids is the fact that normally the interactions governing spin and orbital degrees of freedom have very different energy scales^8^,^9^,^10^. Consequently those degrees of freedom are decoupled at sufficiently low temperatures, hindering the quantum frustration that is required to entangle orbitals and spins. Very recently, x-ray scattering studies in magnetic honeycomb based BaCuSb_2_O_9_ crystals reported indications of spin-orbital entanglement at low temperature^11^,^12^.

An alternative to identifying crystals where spins and orbtitals are strongly coupled would be instead to create artificial crystals where spin and orbital quantum numbers become interchangeable. Such property appears in magnetic Hamiltonians that display SU(4) symmetry^13^. Recent experiments with cold atoms reported spectroscopic quantum simulations in small artificial magnetic systems with SU (*N* ≤ 10) symmetry at ultra low temperature^14^,^15^. Mott physics with SU(2) spins has been observed in optical lattices with ultra cold atoms inside a parabolic potential^16^. In those systems, strong correlations emerge only at extremely low temperatures, making a possible detection of quantum spin-liquids challenging^17^. Solid-state systems where antiferromagnetic interactions have SU(4) symmetry are not common, since in real materials, anisotropies and off-diagonal hopping matrix elements in the degenerate orbital space usually lower that symmetry^18^.

We propose a solid-state system that can be experimentally designed with scanning tunneling microscopy (STM) tips by positioning Coulomb impurity adatoms in a periodic array on top of an insulating honeycomb substrate. The electrons in those substrates can be described by massive Dirac fermions, which form bound states around the impurities^19^,^20^,^21^. Those bound states have spin and valley degeneracies, which are dual to spin-orbital degrees of freedom. We theoretically construct an artificial lattice where each impurity site is quarter filled with valley and spin polarized states. The problem has an emergent SU(4) symmetry that follows from the orthogonality between the two different valley spaces. In systems like graphene, SU(4) symmetry is known to emerge in the quantum Hall regime^22^. Electronic interactions lead to a variety of broken symmetry states in both spins and valleys^23^,^24^,^25^,^26^,^27^,^28^.

The spin-orbital exchange interactions are calculated in three different impurity lattice geometries: triangular, square and honeycomb, shown in Fig. 1. We find the constraints on the impurity lattice in the regimes where the system is expected to behave as a Mott insulator dominated by antiferromagnetic interactions between sites. We propose the experimental conditions for the observation of those states. For honeycomb substrates such as graphene grown on SiC^29^,^30^, we show that the Mott regime of entangled spins and orbitals is experimentally accessible and that the superexchange interaction can be as large as *J*_*s*_/*k* ~ 60–120 K. The experimental signatures of strongly correlated states are discussed based on possible scenarios predicted for SU(4) spin-orbital models^31^,^32^,^33^,^34^, including quantum spin-orbital liquids.

## Results

### Coulomb impurity problem

The wavefunction of the Coulomb impurity bound states for 2D massive Dirac fermions, Ψ(**r**), can be derived from the Dirac equation


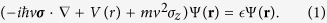


***σ*** = (*σ*_*x*_, *σ*_*y*_) is a vector with off-diagonal Pauli matrices, *σ*_*z*_ is the diagonal Pauli matrix, *v* is the Fermi velocity and *m* is the mass term of the substrate, that describes a gap in the electronic spectrum, Δ = 2 *mv*^2^. 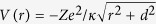 is the Coulomb impurity potential, where *Z* is the atomic number of the impurity, *e* is the electron charge, *κ* the dielectric constant of the surface, and *d* ≈ 2–3 Å is the out-of-plane distance between the impurity and the plane of the substrate.

The impurity potential decays as *V*(*r*) ~ 1/*r* in the 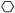 limit and saturates into a constant in the opposite limit. The potential can be written as


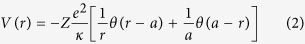


where *a* is an effective real space cut-off which regularizes the Coulomb potential. The size of the cut-off can be chosen as *a* ~ *d* and is typically of the order of the impurity size. This regularization procedure is well known in quantum electrodynamics in 3 + 1 dimensions (QED_3+1_) and has been successfully used to explain the experimentally observed dive of bound states in the lower continuum around super-heavy nuclei with atomic number *Z* > 137^35^,^36^. Both in QED_3+1_ as in the 2D case, the wavefunction of the Coulomb impurity bound states decay over a characteristic distance defined by the Compton wavelength *λ*_*C*_ = *ħ*/*mv*.

In cylindrical coordinates, the solution of Eq. (1) is in the form


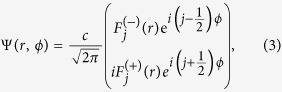


where 
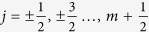
 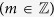 are the possible angular momentum states, and *c* is the normalization constant. The energy spectrum is quantized by the usual quantum numbers in the Hydrogen atom problem, 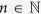 and *j*^19^,^20^,^21^. The degeneracy of the ±|*j*| angular momenta states for a given *n* > 0 however is lifted. At *n* = 0, only the 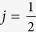 state is allowed.

**Figure 2:**
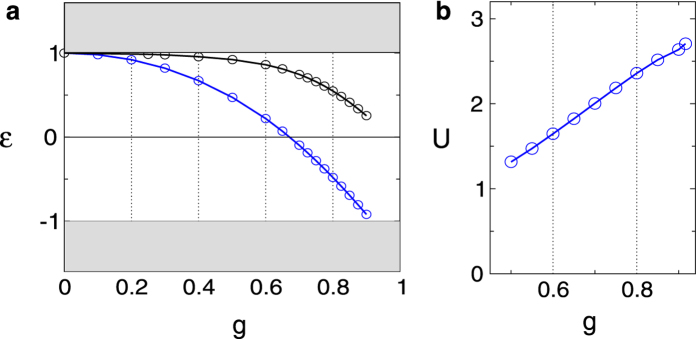
Single impurity energy scales. (**a**) Energy of the Coulomb impurity bound states *ϵ*, in units of *mv*^2^ = 0.13 eV, as a function of the dimensionless coupling *g* = *Zα*. Blue dots: ground state level, *n* = 0, 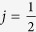. Black dots: first excited state, *n* = 1, 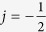. At *g* = *g*_*c*_ ≈ 0.916, the lowest energy level dives in the continuum of negative energy states at *ϵ* = −*mv*^2^. In the subcritical regime 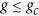, the two levels have an energy separation ~*mv*^2^. (**b**) Hubbard *U*, in units of *mv*^2^*α*, versus *g* in the strong coupling regime 0.5 ≤ *g* ≤ *g*_*c*_. *U* is comparable to the energy of the gap Δ = 2 *mv*^2^.

Defining the impurity strength by the dimensionless coupling *g* ≡ *Zα*, where *α* = *e*^2^/*κħv* is the screened fine structure constant of the substrate, there are two known regimes of the problem: the perturbative regime 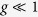, where the bound states are shallow, and the strong coupling regime 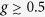, where they dive in the negative sector of the energy spectrum, as shown in Fig. 2. At fixed *g*, the lowest energy level is the *n* = 0, 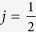 state, followed by the first excited state *n* = 1, 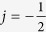. There is an infinite number of higher excited states inside the gap Δ. The latter states have very small binding energies and are not relevant to this discussion.

We are interested in the strong coupling regime of the problem 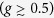, where the confining potential is deep and the energy separation between the ground state level and the first excited state is of the order of ~Δ/2. At sufficiently large coupling, *g* > *g*_*c*_, the lowest energy state level dives in the continuum of negative energy states outside of the gap. This regime is known as the supercritical regime. At the critical one, when *g* = *g*_*c*_ the energy of the lowest level is exactly at the edge of the gap, *ϵ* = −*mv*^2^. In the subcritical regime, 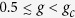, which is the focus of this paper, the levels are strongly localized and sharply defined inside the gap. For a Coulomb impurity on top of graphene epitaxially grown on SiC, where Δ ~ 0.26 eV^29^, and for a typical small distance cut-off *a* ≈ 2.8 Å, *g*_*c*_ = 0.916. In general, the critical coupling *g*_*c*_ ~ 1. The energy of the levels follows directly from matching the wave function at *r* = *a*, similarly to the procedure in the QED_3+1_ case. The solution of the subcritical regime can be calculated either numerically^19^ or for the purposes of this work, analytically, as detailed in the Supplemental Materials.

### Impurity lattice model

In a honeycomb lattice with massive Dirac fermions, the quasiparticles also have two valley flavors, in addition to the spin. The Coulomb impurity bound states therefore must have both spin and valley degrees of freedom. The Dirac equation in this case is


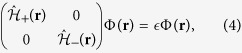


where 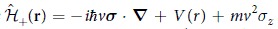 is the Dirac Hamiltonian matrix in valley + and 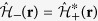 in the opposite valley. The eigenvectors are the four component spinors Φ_*j*,+_(**r**) = (Ψ_*j*_(**r**), **0**) and 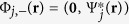, which are degenerate. The *j*-th energy level is four-fold degenerate, with two spins and two valleys. The valleys describe the orbital motion of an electron around a Coulomb impurity. They effectively behave as a pseudo-spin with SU(2) symmetry, as the actual spins.

Once Coulomb interactions among the electrons in the bound state are included, those states tend to spin and valley polarize due to correlations and Pauli blocking. In the ground state, 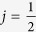, the Coulomb interaction can be expressed in terms of a Hubbard *U* term


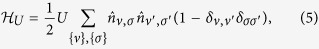


where


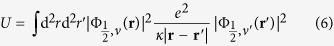


is a valley independent local repulsion. 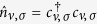 is the number operator per valley and spin at the bound state, where *c*_*ν*,*σ*_ annihilates one electron in the 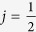 level on valley *ν* with spin *σ*. Due to the orthogonality of the eigenspinors, 
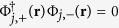
, the exchange interaction between electrons in different valleys around the same Coulomb impurity is zero.

In Fig. 2, we calculate *U* as a function of the dimensionless impurity coupling *g* in the strong coupling regime 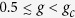. At *g* = *g*_*c*_, *U* = 2.7 *mv*^2^*α*, dropping to *U* = 1.35 *mv*^2^*α* at *g* = 0.5. When *U* is large and only the 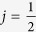 level is filled, the ground state will be singly occupied in one of the four possible states: | 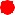 〉 = |+, ↑〉, | 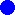 〉 = |+, ↓〉, | 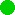 〉 = |−, ↑〉, and | 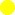 〉 = |−, ↓〉.

We would like to write down an effective lattice model for a strongly correlated lattice of Coulomb impurities, each one having a quarter filled bound state in one of the four possible states above. Those electrons can hop between different Coulomb impurity sites, with each one having a Hubbard *U* energy, that penalizes multiply occupied sites, and also having a well defined valley and spin. The hopping term is


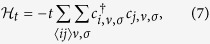


with *c*_*i*_ describing the annihilation operator of an electron in the 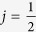 level siting on an impurity site located at **R**_*i*_, and 〈*ij*〉 denotes summation over nearest neighbor (NN) sites. The hopping parameter of the Coulomb impurity lattice is


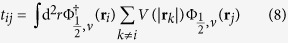


where **r**_*i*_ ≡ **r** − **R**_*i*_ is the position relative to site *i*. Hopping between Coulomb impurity sites conserves valley due to the orthogonality of eigenspinors in the valley space, 
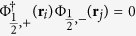
. Because of the summation of the potential over lattice sites and the long range nature of the Coulomb interaction, the value of *t* is influenced by the geometry of the lattice.

In the limit 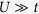, we can expand the effective Hamiltonian in second order perturbation theory in the hopping, 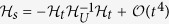. The Hamiltonian that results is the superexchange interaction, which favors antiferromagnetic alignment of spins or valleys. This interaction is of order *J*_*s*_ = *t*^2^/*U* and lowers the energy cost for electrons to hop back and forth between two NN sites. The superexchange competes with the exchange interaction between NN sites, which is ferromagnetic and defined by 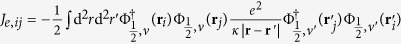, with *J*_*e*,〈*ij*〉_ ≡ *J*_*e*_ < 0. As shown in the Methods section, both the superexchange and the exchange interactions map into a Kugel-Khomskii type Hamiltonian^37^ with *exact* SU(4) symmetry,


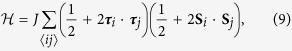


where 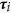 is the valley pseudospin operator and **S**_*i*_ the spin operator on a given site. Hamiltonian (9) is symmetric under any permutation among the four different valley-spin flavors (colors).

The coupling *J* ~ *J*_*s*_ > 0 in the regime where the superexchange coupling dominates 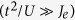. The superexchange interaction is antiferromagnetic, and can drive the spin-orbital lattice into frustrated phases where no symmetry is broken. In the opposite regime 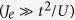, the coupling *J* = −*J*_*e*_ < 0 changes sign, and the system tends to order in a ferromagnetic state at zero temperature.

### Numerical results

**Figure 3:**
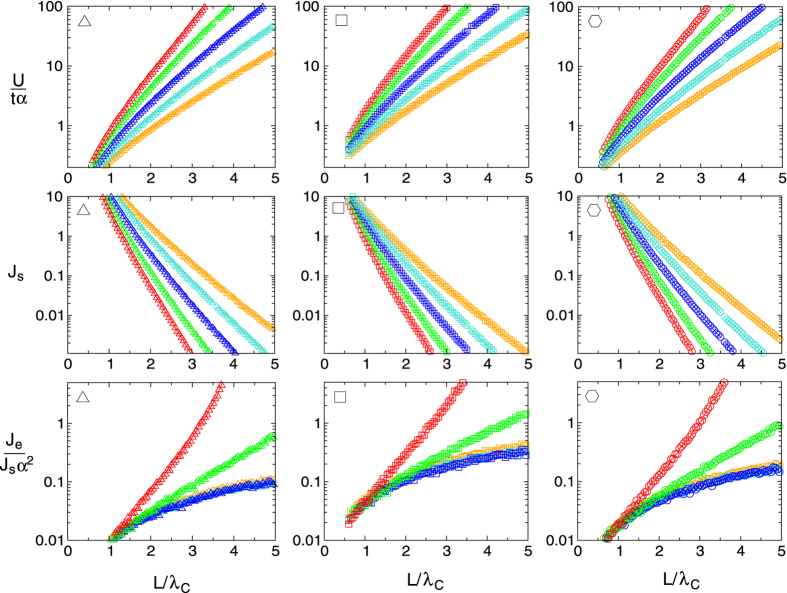
Correlations in Coulomb impurity lattices. Left column 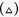: triangular lattice; middle column 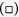 square lattice; right column (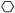): honeycomb lattice. Red dots: *g* = 0.9; green: *g* = 0.8; blue: *g* = 0.7; cyan: *g* = 0.6; orange: *g* = 0.5. Top row: ratio between the onsite repulsion (*U*) and the kinetic energy (*t*) times the fine structure *α* versus the superlattice constant *L* normalized by the Compton wavelength *λ*_*C*_ = *ħ*/*mv*. For a substrate with a gap of of Δ = 0.26 eV (graphene on SiC), *λ*_*C*_ ≈ 46 Å. When 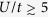, the system is strongly correlated: the Coulomb impurities form a lattice of local spin-orbitals. Middle row: Superexchange interaction, *J*_*s*_ = *t*^2^/*U* in units of *mv*^2^/*α*, versus *L*/*λ*_*C*_. Bottom row: ratio between the exchange interaction *J*_*e*_ and the superexchange interaction *J*_*s*_ times *α*^2^.

In Fig. 3 we show the ratio of *U*/*tα* as a function of the impurity lattice constant *L* for three different geometries: triangular 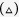, square 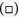 and honeycomb (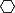). *L* is normalized by the Compton wavelength *λ*_*C*_, which is inversely proportional to the mass gap of the substrate. In the regime where 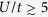, the system is a strongly correlated insulator and can be effectively described as a lattice of local valley-orbitals and spins. The different curves in each panel correspond to different impurity couplings, with *g* ranging from 0.5 to the critical value *g*_*c*_ ~ 0.916. At the middle row panels, we display the superexchange coupling *J*_*s*_ (in units of *mv*^2^/*α*) as a function of *L*. For couplings *g* < *g*_*c*_, when *U*/*tα* ~ 12 the superexchange coupling ranges from *J*_*s*_*α*/*mv*^2^ ≈ 0.01–0.02 for *g* running between 0.5 and 0.9 in all geometries we tested, as indicated in Fig. 3. In the regime *U*/*tα* ~ 20, the super exchange is in the range *J*_*s*_*α*/*mv*^2^ ≈ 0.003–0.007.

For graphene on SiC substrate with Δ = 2 *mv*^2^ ~ 0.26 eV, the Compton wavelength *λ*_*C*_ ≈ 46 Å. On the surface of SiC (*κ* ~ 5.2) the fine structure constant *α* ≈ 0.42. The size of the superlattice constant *L* that corresponds to a fixed value of *J*_*s*_ varies slightly depending on the geometry of the lattice. At *g* ≈ *g*_*c*_ (red dots), the impurity valence *Z* ~ 2. When *U*/*tα* = 12 (*U*/*t* ≈ 5), the superexchange interaction between NN sites is *J*_*s*_/*k* ~ 59 K and corresponds to impurity lattice constants *L*/*λ*_*C*_ ≈ 2.25 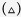, 1.9 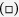, and 2.1 (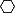), resulting in *L* ~ 90–100 Å. At *g* = 0.5 or *Z* ~ 1 (orange dots), the wavefunctions are more weakly bounded to the impurities and hence more extended. The same ratio of *U*/*t* ≈ 5 corresponds to *J*_*s*_/*k* ~ 28 K and larger superlattice constants *L*/*λ*_*C*_ ~ 4.6 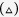, 3.9 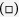, and 4.3 (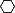), respectively, with *L* ~ 180 Å–200 Å. For a larger gap of Δ ~ 0.5 eV^30^, the superexchange nearly doubles (*J*_*s*_ ~ 56–118 K) while the Compton wavelength is halved. When *U*/*tα* = 20 (*U*/*t* ≈ 8.5), *J*_*s*_/*k* ~ 10–20 K.

In the regime of interest, where *U*/*t* is large, *U* is the largest energy scale in the problem. The superexchange interaction competes with the exchange one *J*_*e*_ and, in principle, both can be of the same order. In the bottom row of the panels in Fig. 3 we plot the ratio between *J*_*e*_/*J*_*s*_*α*^2^. For *α* < 1, the superexchange interaction clearly dominates the exchange interaction, and is at least three times larger for 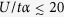. When considering Coulomb impurities on graphene-SiC substrates, where *α* = 0.42, the ratio *J*_*e*_/*J*_*s*_ < 0.07 in all geometries considered in the range 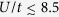. The dominant interactions are therefore clearly antiferromagnetic. Due to the SU(4) symmetry, valley and spin degrees of freedom are strongly entangled and may give rise to a spin-orbital liquid in the Mott insulator regime.

### Experimental setup

The lattice of Coulomb impurities can be experimentally created with STM tips, which can drag atoms on a surface with atomic precision^38^. Possible substrates include graphene epitaxially grown on SiC, which was shown to develop a gap ranging from Δ = 0.26–0.5 eV^29^,^30^. In high quality samples, the Fermi level was observed in the middle of the gap^30^. Other crystals, such as MoS_2_, MoSe_2_, and other dichalcogenides^39^, have even larger gaps, however they also exhibit large spin-orbit couplings^40^, which will lift the SU(4) symmetry of the problem, lowering it to SU(2). Strong unitary disorder connects the two valleys and can also have a similar effect. Disorder effects, however, can be inhibited by properly annealing the substrate.

Among alkaline metals, potassium adatoms (*Z* = 1) are known to spontaneously form two dimensional crystals on honeycomb substrates such as graphite^41^. Higher valence cobalt adatoms have already been studied with STM on graphene and are also possible candidates^42^. The strong coupling regime, where the bound states are deep and well separated, is experimentally accessible for impurities with a valence *Z* ~ 1. That contrasts with the standard relativistic scenario, where the strong coupling regime can be achieved only when the valence is of the order of the inverse of the QED fine structure constant *Z* ~ 1/*α*_*QED*_ = 137.

The determination of the impurity lattice constant *L* that is required to create a Mott insulator with strong antiferromagnetic correlations can be achieved with local spectroscopy measurements around a single impurity. Those measurements can accurately determine the energy of the bound states inside the gap. With the theoretical wavefunctions, one can extract the effective impurity coupling *g* by comparing the measurement of the energy levels with the calculated result, as shown in Fig. 2. The appropriate range for the impurity lattice constant is indicated in the plots of Fig. 3. Integration of the measured local density of states over the area around the impurity gives the occupation of the ground energy level inside the gap. When the impurity lattice is in the Mott regime, each four-fold degenerate impurity level will remain singly occupied (quarter filling).

## Discussion

**Figure 4:**
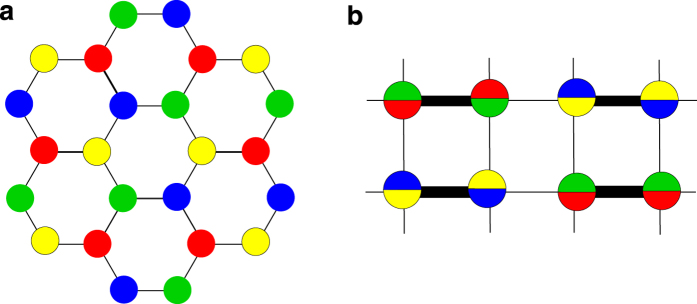
Spin-orbital color states. (**a**) Possible algebraic quantum spin-orbital liquid for the honeycomb lattice in the SU(4) Heisenberg model, numerically predicted in ref. 31. This state may correspond to a quarter filled *π*-flux phase. Each color is surrounded by the same states, up to color permutations. Both crystalline and SU(4) symmetries are intact. (**b**) Possible dimerized state in the square lattice, with alternating singlets of two colors (after ref. 33). This state has long range order and breaks both lattice and color symmetry.

Recent numerical evidence^31^ suggests that the ground state of the antiferromagnetic Hamiltonian (9) in the honeycomb lattice is a strongly correlated state that preserves all the symmetries of the system. This state is a quantum spin-orbital liquid schematically drawn in the left panel of Fig. 4. Every site has a well defined spin-valley state (color) among the four possible colors. Each color has the same neighbors up to color permutations. The pattern preserves both the lattice symmetry and the SU(4) color symmetry.

Color-color correlations appear to decay as a power law, indicating a gapless state, or equivalently, an algebraic quantum spin-orbital liquid with no true long range order. Algebraic spin liquids are generally known to be robust two-dimensional interacting critical states, relevant to a variety of correlated physical models^43^. After comparison of the energy of several different states, the quarter filled *π*-flux state currently appears as the leading candidate^31^,^44^. In the honeycomb lattice, a *π*-flux in the honeycomb plaquette creates Dirac fermions at quarter filling, which is the regime of interest for Mott insulators with SU(4) symmetry. Those Dirac fermions are (color) spinon excitations, which are four-fold degenerate due to the color symmetry.

Low-energy characteristic probes amenable to 2D systems have been proposed, such as injecting a spin current into the insulator and monitoring the spin bias dependence of the current^46^. In the simplest experimental setup with a single metal-insulator interface, spin accumulation is achieved via the spin Hall effect. In the four-terminal setup, the spin-liquid insulator is coupled to left and right metal leads. Spin current detection occurs through the reverse spin Hall effect in one of the metallic contacts.

In the spin-orbital (valley) case at hand, the spin degrees of freedom in the insulator and in the metal are coupled at the interface. The valleys are decoupled from the orbital degrees of freedom in the metal. Hence the valleys do not experience flips due to the spin current injection. The result is the propagation of a pure spin current with additional valley degeneracy. Consequently, in this case, the spin current will scale in the same way with the bias voltage as in pure spin models. For the *π*-flux state, the Dirac cone of the spinons is degenerate in all quantum numbers (spin and valley). The spin current scales with the fifth power of the bias voltage, *I*_*s*_ ~ *V*^5 ^45,46. This result appears to be a universal signature of both spin and spin-orbital liquid phases with gapless Dirac fermion spinons. In general, the power of the spin voltage dependence is sensitive to the nature and dispersion of the spinon excitations. The exact nature of the spin-orbital liquid state in the honeycomb lattice requires further investigation. Nevertheless, the prospects of observing a true quantum spin-orbital liquid in this geometry seem quite promising.

Triangular lattices are natural candidates for quantum disordered states due to their strongly frustrated nature. It was proposed at first that their ground state has plaquette order^13^, with plaquettes formed by SU(4) singlets. However more recent work^32^ found strong local resonances between plaquette configurations. While more complicated orders with large unit cells can not be ruled out, the ground state appears to be a spin-orbital liquid with no plaquette order. The presence of next-nearest neighbor superexchange 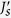 drives the system into magnetically long range ordered state via a quantum phase transition at a critical value 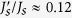
32. In the proposed Coulomb impurity lattice, we find that ratio to be ~10^−2^. On the basis of the existing knowledge about the model, we conclude that a spin-orbital liquid state can be realized in the Mott regime. The nature of this state is not yet known.

There have been suggestions of a variety of different ground states for Hamiltonian (9) in the square lattice. Possibilities include a gapless spin liquid with nodal fermions^34^, and a plaquette state^13^,^47^. A more recent numerical work has laid more concrete evidence towards a dimerized state depicted in Fig. 4, which breaks both lattice and color symmetry^33^. The thick bonds represent strong bonds, while the think lines are weaker. This particular state has two sets of dimers with two colors each, which alternate along the two main directions of the lattice. Because of the broken symmetry, the elementary excitations are Goldstone modes in the form of gapless (color) magnons. These could also lead to characteristic power law dependencies in the spin current as a function of spin bias^45^,^46^, with the power being generally smaller than for gapless Dirac spinons (*π*-flux phase).

Coulomb impurity lattices offer wide possibilities for different frustrated scenarios due to the inherent flexibility in their design. Recent experiments observed evidence for a spin-liquid ground state in the antiferromagnetic Kagome lattice^48^. We conjecture that gapped honeycomb substrates with large spin orbit coupling, such as MoS_2_^40^, could be experimentally used to design frustrated artificial Coulomb impurity lattices where the spin degeneracy is explicitly lifted, leaving a pure quantum orbital (valley) liquid in the ground state. The tendency towards frustration is not the unique scenario for artificial lattices supported on gapped honeycomb substrates. For instance, color ferromagnetism is possible in superlattices of mass defects forming quantum rings^49^.

In summary, we have shown that Mott insulators having spin and orbital degeneracies can be artificially designed in a solid state system. The emergent SU(4) symmetry of the problem follows from the unique nature of the valley degrees of freedom in honeycomb substrates and does not require fine tuning. We have predicted the conditions for Coulomb impurity lattices to be in the Mott regime and discussed experiments that could detect quantum spin-orbital liquid states.

Most of the current efforts to simulate quantum spin liquids are concentrated in cold atom systems, where the Mott physics is present only at ultra low temperatures^16^,^17^. This proposal may lead to significant advances in the experimental design and observation of quantum spin-orbital liquids in solid-state settings.

## Methods

### Wavefunctions

We assume a real space cut-off for the Coulomb interaction *a* = *λ*_*C*_/18. For a typical mass gap energy *mv*^2^ ≈ 0.13 eV and *ħv* ≈ 6 eV Å, as in graphene on SiC, the Compton wavelength *λ*_*C*_ ~ 50 Å, which corresponds to *a* ≈ 2.8 Å. This number agrees with the typical size of many Coulomb impurities, including alkaline metals.

The analytical form of the 2D Coulomb impurity wavefunctions in the weak coupling regime 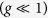 is well known^20^,^21^. In that regime the cutoff does not play an important role (can be set to zero) and the bound states are shallow. The wavefunctions in the subcritical strong coupling regime 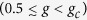 can be solved analytically as well. They correspond to the solution of the Dirac equation in the potential (2) and bare strong similarity to the 3D Dirac equation (QED_3+1_) case^35^,^36^.

Setting *ħ* = *v* = 1, for *r* > *a*, the strong coupling solution in the subcritical regime has spinor component amplitudes


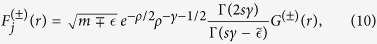


where 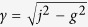, 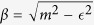, 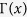 is a gamma function and


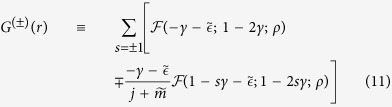


is defined in terms of confluent hypergeometric functions of the first kind. 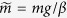, 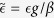 and 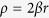 are the normalized mass, energy and distance away from the impurity (see supplemental materials). For *r* ≤ *a*, the solution is defined in terms of Bessel functions


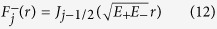


and


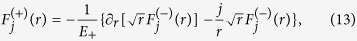


where *E*_±_ = *ϵ* − *V*(*a*) ± *m*.

The energy of the levels follows from matching the wavefunctions at *r* = *a*, Ψ_*r*<*a*_(*a*) = Ψ_*r*>*a*_(*a*), as shown in Fig. 2. For a given angular momentum state *j*, there is an infinite number of solutions that can be labeled by the index 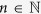, which is a non-negative integer. The lowest energy solution is labeled *n* = 0, with higher *n* > 0 attributed to the other higher excited states. For 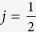 and *ϵ* = −*m*, the critical coupling of the *n* = 0 level state is *g*_*c*_ = 0.916. The spectrum is in excellent agreement with the numerical results of ref. 19.

### Hubbard *U* term

The Coulomb interaction among electrons in the lowest energy state *n* = 0 and 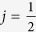 is described by


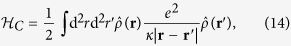


where 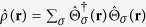 is the density operator defined in terms of the field operator 
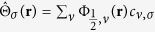
. Hamiltonian (14) can be expressed explicitly in terms of *c* operators, resulting in the Hubbard *U* Hamiltonian described in the main text. The exchange term that also follows from (14) is identically zero due to the orthogonality of the two valley eigenspinors.

### Spin-orbital exchange Hamiltonian

In second order of perturbation theory, the superexchange Hamiltonian is expressed in terms of *c* operators as:


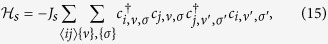


with *J*_*s*_ = *t*^2^/*U*. The exchange interaction between NN sites can be calculated from the Coulomb interaction 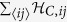,


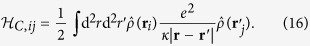


We extend the definition of the field operators as a sum over lattice sites, 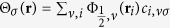. The exchange part of the interaction above term can be explicitly written as


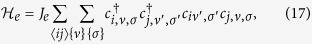


where *J*_*e*_ is given in the text. Hamiltonians (15) and (17) both map into pseudospin (valley) and spin operators, 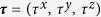 and **S** = (*S*^*x*^, *S*^*y*^, *S*^*z*^), through the following relations:


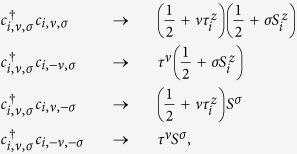


where 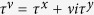 and *S*^*σ*^ = *S*^*x*^ + *σiS*^*y*^. *ν* = ±, and *σ* = ± index the two valleys and spins respectively. This mapping results in Hamiltonian (9).

## Additional Information

**How to cite this article**: Dou, X. *et al.* Designing Quantum Spin-Orbital Liquids in Artificial Mott Insulators. *Sci. Rep.* **6**, 31737; doi: 10.1038/srep31737 (2016).

## Supplementary Material

Supplementary Information

## Figures and Tables

**Figure 1:**
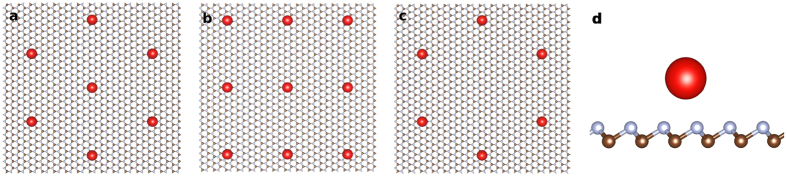
Coulomb impurity lattices. Honeycomb substrate with unequal sublattices decorated with a superlattice of charged impurities. In the three configurations, triangular (**a**), square (**b**) and honeycomb (**c**), the impurities are separated by a superlattice constant *L*, and sit at a distance *d* away from the plane of the substrate (**d**). All impurities interact with electrons via Coulomb, 1/*r* potential.
