# Supplementary material for: Designing Quantum Spin-Orbital Liquids in Artificial Mott Insulators
Source: Sci Rep. 2016 Aug 24;6:31737. doi: 10.1038/srep31737 (PMC4995463; doi:10.1038/srep31737)
Supplement: Supplementary Information [file srep31737-s1.pdf]

Suplementary Materials of "Designing Quantum Spin-Orbital Liquids in Artificial Mott Insulators"

Xu Dou,[1] Valeri N. Kotov,[2] and Bruno Uchoa[1,*]

[1]*Department of Physics and Astronomy, University of Oklahoma, Norman, OK 73069, USA*
[2]*Department of Physics, University of Vermont, Burlington, VT 05405, USA*

Wavefunction of the strong coupling subcritical regime

The wave function $\Psi(\mathbf{r})$ of a two dimensional massive Dirac fermion moving around a Coulomb impurity satisfies,

$$(-i\boldsymbol{\sigma}\cdot\boldsymbol{\nabla}+V(r)+m\sigma_z)\Psi(\mathbf{r})=\epsilon\Psi(\mathbf{r}), \tag{1}$$

where

$$V(r)=\begin{cases}-g/r, & r>a\\ -g/a, & r\leq a\end{cases}$$

is the regularized Coulomb potential. Here we set $\hbar=v=1$.

The two-component wave function is

$$\Psi(r,\phi)=\frac{1}{\sqrt{2\pi}}\begin{pmatrix}F_j^{(-)}(r)e^{i(j-1/2)\phi}\\ F_j^{(+)}(r)e^{i(j+1/2)\phi}\end{pmatrix}, \tag{2}$$

he Dirac equation with the presence of an external potential becomes

$$\begin{bmatrix}\epsilon-m-U & -(\partial_r+\frac{\kappa+1}{r})\\ (\partial_r-\frac{\kappa}{r}) & \epsilon+m-U\end{bmatrix}\begin{pmatrix}F_j^{(-)}(r)\\ F_j^{(+)}(r)\end{pmatrix}=0$$

or equivalently

$$\begin{aligned}\frac{dF_j^{(-)}}{dr}-\frac{\kappa}{r}F_j^{(-)}+(\epsilon+m-U)F_j^{(+)}=0\\ \frac{dF_j^{(+)}}{dr}+\frac{\kappa+1}{r}F_j^{(+)}-(\epsilon-m-U)F_j^{(-)}=0\end{aligned} \tag{3}$$

where $\kappa=j-\frac{1}{2}$.

Solution for $r>a$

The wave functions assumes the form (j indexes will be dropped)

$$F^{(-)}(\rho)=\sqrt{m+\epsilon}e^{-\rho/2}\rho^{\gamma-1/2}(Q_1+Q_2)$$

$$F^{(+)}(\rho)=\sqrt{m-\epsilon}e^{-\rho/2}\rho^{\gamma-1/2}(Q_1-Q_2). \tag{4}$$

where $\rho=2\lambda r$, $\beta=\sqrt{m^2-\epsilon^2}$, and$\gamma=\sqrt{j^2-g^2}$. After some algebra, we get

$$\rho Q_1'+(\gamma-\frac{\epsilon g}{\beta})Q_1-(j+\frac{mg}{\beta})Q_2=0 \tag{5}$$

$$\rho Q_2'+(\gamma+\frac{\epsilon g}{\beta}-\rho)Q_2-(j-\frac{mg}{\beta})Q_1=0. \tag{6}$$

These equations can be decoupled

$$\rho Q_1'' + (1+2\gamma-\rho)Q_1' - (\gamma - \frac{\epsilon g}{\beta})Q_1 = 0$$

$$\rho Q_2'' + (1+2\gamma-\rho)Q_2' - (1+\gamma - \frac{\epsilon g}{\beta})Q_2 = 0,$$

both of which are Kummer's differential equation

$$xy'' + (c-x)y' - ay = 0$$

with the solution $y = c\mathcal{F}(a;c;x)$. Here we call the confluent Hypergeometric function of the first kind ${}_1F_1(a;c;x)$ as $\mathcal{F}(a;c;x)$,

$${}_1F_1(a;c;x) = 1 + \frac{a}{c}x + \frac{a(a+1)}{c(c+1)}\frac{x^2}{2!} + ...,$$

and notice that ${}_1F_1(a;c;x=0) = 1$. The solutions are

$$Q_1 = c_1\mathcal{F}(\gamma - \frac{\epsilon g}{\beta}; 1+2\gamma; \rho) + d_1\mathcal{F}(-\gamma - \frac{\epsilon g}{\beta}; 1-2\gamma; \rho) \tag{7}$$

$$Q_2 = c_2\mathcal{F}(1+\gamma - \frac{\epsilon g}{\beta}; 1+2\gamma; \rho) + d_2\mathcal{F}(1-\gamma - \frac{\epsilon g}{\beta}; 1-2\gamma; \rho). \tag{8}$$

Weak coupling regime

When $g < j$, γ is real. Integrability of the wavefunction at $\rho \to \infty$ requires that $d_1 = d_2 = 0$. From Eq.(5), one can determine the ratio

$$\frac{c_1}{c_2} = \frac{Q_1}{Q_2}\bigg|_{\rho=0} = \frac{j + \frac{mg}{\beta}}{\gamma - \frac{\epsilon g}{\beta}}.$$

To simplify the notation we call $\tilde{m} = mg/\beta$, $\tilde{\epsilon} = \epsilon g/\beta$, and

$$c_1 = c,\ c_2 = \frac{\gamma - \tilde{\epsilon}}{j + \tilde{m}}c.$$

That leads to the solution

$$F_j^{(-)}(\rho) = c\sqrt{m+\epsilon}e^{-\rho/2}\rho^{\gamma-1/2}[\mathcal{F}(\gamma-\tilde{\epsilon};1+2\gamma;\rho) + \frac{\gamma-\tilde{\epsilon}}{j+\tilde{m}}\mathcal{F}(1+\gamma-\tilde{\epsilon};1+2\gamma;\rho)]$$

$$F_j^{(+)}(\rho) = c\sqrt{m-\epsilon}e^{-\rho/2}\rho^{\gamma-1/2}[\mathcal{F}(\gamma-\tilde{\epsilon};1+2\gamma;\rho) - \frac{\gamma-\tilde{\epsilon}}{j+\tilde{m}}\mathcal{F}(1+\gamma-\tilde{\epsilon};1+2\gamma;\rho)]$$

This solution is regular at $\rho \to 0$, and the short distance cut-off can be set to zero.

Strong coupling regime

When the coupling $g > \frac{1}{2}$, γ becomes imaginary. With the requirement of imposing a small distance cut-off, the condition that the wave function behaves well at $\rho = 0$ is not necessary, so we should include both $\pm\gamma$ branches into the solutions. The ratio between the two And in this case we hope the wave functions die off at $\rho \to +\infty$, which

can also serve to settle down the ratio between $\gamma-$branch and $(-\gamma)$-branch. The formula can be used here is the asymptotic form of the hypergeometric function,

$$ {}_1F_1(a;b;x) \sim \Gamma(b)\left(\frac{e^z z^{a-b}}{\Gamma(a)} + \frac{(-z)^{-a}}{\Gamma(b-a)}\right). $$

The second part is required if the gamma function $\Gamma(a)$ is infinite (when a is a negative integer) or $\mathrm{Re}(z)$ is non-positive. In our case, we could exclude these two conditions, and only keep the second term. Therefore for large $|z|$, the dominating part (which is growing) of $\mathcal{F}(z)$ is

$$ \mathcal{F}(a;b;z) \sim \frac{\Gamma(b)}{\Gamma(a)} e^z z^{a-b}, $$

and we ask for some condition to cancel this term. For $aF(\rho;\gamma) + bF(\rho;-\gamma)$ we need the following two terms to be finite at $\rho \to +\infty$

$$ a\rho^{\gamma-1/2}\mathcal{F}(\gamma-\tilde{\epsilon};1+2\gamma;\rho) + b\rho^{-\gamma-1/2}\mathcal{F}(-\gamma-\tilde{\epsilon};1-2\gamma;\rho) \tag{9} $$

$$ a\rho^{\gamma-1/2}\frac{\gamma-\tilde{\epsilon}}{j+\tilde{m}}\mathcal{F}(1+\gamma-\tilde{\epsilon};1+2\gamma;\rho) + b\rho^{-\gamma-1/2}\frac{-\gamma-\tilde{\epsilon}}{j+\tilde{m}}\mathcal{F}(1-\gamma-\tilde{\epsilon};1-2\gamma;\rho) \tag{10} $$

From 9,

$$ \frac{a}{b} = -\frac{\Gamma(\gamma-\tilde{\epsilon})}{\Gamma(1+2\gamma)}\frac{\Gamma(1-2\gamma)}{\Gamma(-\gamma-\tilde{\epsilon})} = -\frac{\Gamma(\gamma-\tilde{\epsilon})}{(2\gamma)\Gamma(2\gamma)}\frac{(-2\gamma)\Gamma(-2\gamma)}{\Gamma(-\gamma-\tilde{\epsilon})} = \frac{\Gamma(\gamma-\tilde{\epsilon})}{\Gamma(2\gamma)}\frac{\Gamma(-2\gamma)}{\Gamma(-\gamma-\tilde{\epsilon})}. $$

We can assign

$$ a = \frac{\Gamma(-2\gamma)}{\Gamma(-\gamma-\tilde{\epsilon})}, \qquad b = \frac{\Gamma(2\gamma)}{\Gamma(\gamma-\tilde{\epsilon})} \tag{11} $$

The solution for $F^{(\pm)}$ is

$$ \begin{aligned} F_j^{(\mp)}(r) &= c'\sqrt{m\pm\epsilon}\,e^{-\rho/2}\rho^{-1/2} \\ &\times\left[\frac{\Gamma(-2\gamma)}{\Gamma(-\gamma-\tilde{\epsilon})}\rho^{\gamma}\mathcal{F}(\gamma-\tilde{\epsilon};1+2\gamma;\rho) + \frac{\Gamma(2\gamma)}{\Gamma(\gamma-\tilde{\epsilon})}\rho^{-\gamma}\mathcal{F}(-\gamma-\tilde{\epsilon};1-2\gamma;\rho)\right. \\ &\left|\pm\frac{\Gamma(-2\gamma)}{\Gamma(-\gamma-\tilde{\epsilon})}\frac{\gamma-\tilde{\epsilon}}{j+\tilde{m}}\rho^{\gamma}\mathcal{F}(1+\gamma-\tilde{\epsilon};1+2\gamma;\rho) \pm \frac{\Gamma(2\gamma)}{\Gamma(\gamma-\tilde{\epsilon})}\frac{-\gamma-\tilde{\epsilon}}{j+\tilde{m}}\rho^{-\gamma}\mathcal{F}(1-\gamma-\tilde{\epsilon};1-2\gamma;\rho)\right] \end{aligned} \tag{12} $$

Solution for $r \le a$

In the $r < a$ region, we define

$$ F_j^{(-)}(r) = \frac{1}{\sqrt{r}}A(r) $$
$$ F_j^{(+)}(r) = \frac{1}{\sqrt{r}}B(r) $$

the Dirac equation becomes

$$ A'(r) - \frac{j}{r}A(r) + E_+B(r) = 0 $$

$$ B'(r) + \frac{j}{r}B(r) - E_-A(r) = 0 $$

where $E_\pm = \epsilon + \frac{g}{a} \pm m$. These equations can be decoupled into

$$A''(r) + (E_+E_- + \frac{j-j^2}{r^2})A(r) = 0$$

$$B''(r) + (E_+E_- - \frac{j+j^2}{r^2})B(r) = 0$$

The solutions are

$$A(r) = c_1\sqrt{r}J_{j-1/2}(\sqrt{E_+E_-}r)$$

$$B(r) = c_2\sqrt{r}J_{j+1/2}(\sqrt{E_+E_-}r)$$

and $\sqrt{E_+E_-} = \sqrt{\epsilon^2 + (g/a)^2 + (2\epsilon g/a) - m^2}$.

From

$$B(r) = -\frac{A' - \frac{j}{r}A}{E_+},$$

we have

$$B(r) = -\frac{c_1}{E_+}[\frac{\frac{1}{2}-j}{\sqrt{r}}J_{j-1/2}(\sqrt{E_+E_-}r) + \sqrt{r}J'_{j-1/2}(\sqrt{E_+E_-}r)]$$

Energy

The energy ϵ can be determined by matching the inside solution and the outside one, formally through

$$Out(j,\epsilon,r,g)\big|_{r=a} = Ins(j,\epsilon,r,g)\big|_{r=a}$$

For given j, g, and at $r=a$, we can determine the energy ϵ

$$\begin{pmatrix} \sqrt{r}F(r) \\ \sqrt{r}G(r) \end{pmatrix}\Bigg|_{r=a} = \begin{pmatrix} A(r) \\ B(r) \end{pmatrix}\Bigg|_{r=a}$$

* Electronic address: uchoa@ou.edu

[1] D.S. Novikov, Phys. Rev. B 76, 245435 (2007).
[2] V.M. Pereira, V.N. Kotov, and A.H. Castro Neto, Phys. Rev. B 78, 085101 (2008)
